# Polyphenol Bioaccessibility and Sugar Reducing Capacity of Black, Green, and White Teas

**DOI:** 10.1155/2013/238216

**Published:** 2013-04-04

**Authors:** Shelly Coe, Ann Fraser, Lisa Ryan

**Affiliations:** Functional Food Centre, Oxford Brookes University, Gipsy Lane, Oxford OX3 0BP, UK

## Abstract

Tea (*Camellia sinensis*) is a widely consumed beverage and recognised for its potential enhancing effect on human health due to its rich polyphenol content. While a number of studies have investigated the quantity and type of polyphenols present in different tea samples, no study has reported the potential effect of digestive enzymes on the availability of tea polyphenols for human absorption or the subsequent impact on glycaemic response. The objectives of the present study were to assess the total polyphenol content of different teas, to assess the bioaccessibility of polyphenols in whole and bagged teas, and to determine the effect of black, white, and green tea infusions on sugar release. All of the teas were a significant source of polyphenols (10–116 mg Gallic acid equivalents/g). There was an overall increase in the release of polyphenols from both the bagged and the whole teas following *in vitro* digestion. Bagged green tea significantly (*P* < 0.05) reduced rapidly digestible starch from white bread samples compared to control and black and white bagged teas. The present study confirms that tea is a rich source of polyphenols and highlights the potential benefits it may have on modulating glycaemic response in humans.

## 1. Introduction

One of the most widely consumed beverages throughout the world is tea produced from the tea plant (*Camellia sinensis*). Tea for consumption is classified according to the methods used in its production. Geographical consumption patterns of the different teas vary greatly with green tea consumed mainly in Asia and the Middle East and black tea consumed mostly in western countries.

Tea has been found to be a rich source of polyphenols and antioxidants [1]. This together with evidence from epidemiological studies [2] and high consumption rates worldwide has led to growing interest in tea as a product that may significantly contribute to human health.

The polyphenol profile of the different teas is affected by their different methods of production. Black tea is produced by wilting, crushing, and partial oxidation and consequently is rich in theaflavins and thearubigins [3]. In green tea production oxidation is minimised resulting in catechins being dominant [4], particularly epigallocatechin-gallate (EGCG), white tea undergoes the least processing and is produced from young leaves and buds resulting in high levels of EGCG [5], though generally lower than the levels found in green tea.

Reduced risk of coronary heart disease [6], stroke incidence [7], chronic inflammation [8], and cancer incidence [9] is associated with black and green tea consumption. Recent studies have focused on the impact of tea and tea polyphenols on blood glucose (BG) and insulin sensitivity. Black tea has been shown to decrease plasma glucose and enhance insulin concentrations after consumption in comparison to a control and a caffeine drink [10]. Aldughpassi and Wolever [11] showed that 250 mL of black tea with test meals actually increased overall mean peak BG compared to water though a reduction in the standard error might indicate the ability of tea compounds to improve the precision of the BG response. A study of green tea catechins in insulin resistant induced obese rats suggested that they may impact glucose control through several pathways [12].

The objectives of this study were as follows:

1. to assess the total polyphenol content of different commercial teas;
2. to assess the bioaccessibility of polyphenols from whole and bagged teas after *in vitro* digestion;
3. to determine the effect of black, white, and green tea infusions on sugar release from bread after an *in vitro* digestion model.

## 2. Materials and Methods

### 2.1. Chemicals

All chemicals and reagents were of analytical grade and purchased from Sigma-Aldrich (Poole, UK). The different teas were sourced directly from a specialist supplier as whole teas or purchased in bag form from a local Tesco supermarket.

### 2.2. Study Protocol

The weight of each whole tea used was approximately 3 g except for the “Flowering Osmanthus” and “Jasmine & Lily” teas where an entire bulb was used (the weight of each bulb was recorded). For teas in bagged format, one tea bag (approximately 2.5 g) was used. All tea samples were prepared using a standard protocol. Each tea was infused in 200 mL of boiling water (90°C unless otherwise specified on the manufacturer's instructions) for three minutes and then stirred six times before the tea was removed. The resultant sample was then left at room temperature to cool for an additional 17 minutes before testing commenced. All tests were carried out on a minimum of three separate occasions and samples were analysed in triplicate for each test.

### 2.3. Analysis of Polyphenol Content

#### 2.3.1. Folin-Ciocalteu (FCR)

Samples from nineteen brands of commercially available tea were chosen. An aliquot (200 *μ*L) of each tea sample was added to 1.5 mL of freshly prepared Folin-Ciocalteu reagent (1 : 10 v/v with water). The mixture was allowed to equilibrate for 5 min and then mixed with 1.5 mL of 60 g/L sodium carbonate solution. After incubation at room temperature for 90 min, the absorbance of the mixture was read at 725 nm using the respective solvent as blank. The results were expressed as mg of gallic acid equivalents (GAEs) per gram of tea.

### 2.4. Bioaccessibility of Tea Polyphenols

Samples from nineteen brands of commercially available tea were analysed using an *in vitro* digestion model adapted from Ryan and others [13]. A total of 4 mL of each 200 mL tea infusion was added to an amber vial and made up to a volume of 15 mL with saline. A 1 mL baseline aliquot was taken from each sample. The samples were acidified to pH 2 by the addition of 1 mL of a porcine pepsin preparation (0.04 g pepsin in 1 mL 0.1 M HCl) and then incubated at 37°C in a shaking water bath at 95 rpm for 1 hour. Gastric aliquots were taken. The pH was increased to 5.3 with 0.9 M sodium bicarbonate, followed by the addition of 200 *μ*L of the bile salts glycodeoxycholate (0.04 g in 1 mL saline), taurodeoxycholate (0.025 g in 1 mL saline) and taurocholate (0.04 g in 1 mL saline), and 100 *μ*L of pancreatin (0.04 g in 500 *μ*L saline) to each sample. The pH was adjusted to 7.4 using 1 M NaOH and overlaid with nitrogen. The samples were then incubated in a shaking water bath for two hours at 37°C. Duodenal aliquots were taken and samples were frozen until analysis.

### 2.5. Measurement of Sugar Release

The relative glycaemic impact (RGI) of one black tea, one green tea, and one white tea sample from the Clipper brand was measured in order to assess the effect of the tea polyphenols on the inhibition of starch breakdown. Bread was used as the starch source. This was achieved by subjecting samples of tea combined with bread to an *in vitro* digestion procedure and measuring the resultant reducing sugars released.

#### 2.5.1. Bread Preparation

White bread dough was made to a recipe of 190 g warm tap water, 1 tbsp virgin olive oil, 1 tsp salt, 1 tbsp sugar, 1 tbsp dried milk powder, 350 g strong white flour, and 1.5 tsp of yeast. The dough was then baked in a *Russell Hobbs* bread maker (model no: 18036, Manchester, UK) for a total of 3 hours and 20 minutes. Samples of the bread were then prepared by weighing 2.5 g samples and placing each into 60 mL specimen pots. The pots were inserted into an aluminium heating block and covered with an insulating sheet in readiness for testing.

#### 2.5.2. *In Vitro *Digestion

An *in vitro* digestion procedure was used to test the tea samples. This consisted of a simulated gastric digestion phase followed by an ileal digestion phase with timed sampling at the end of the gastric phase and during the ileal phase [14]. A volume of 30 mL of each tea infusion (1 tea bag/infusion) was added to its own individual bread sample. A 250 *μ*L baseline sample was extracted for each sample at *t* = 0 min and added to a test tube in a ratio of 1 : 4 in ethanol. This was followed by the addition to each sample of 0.1 mL 10%  *α*-amylase, 0.8 mL 1 M HCl, and 1 mL 10% pepsin solution in 0.05 M HCl to each. The resultant mixture was stirred slowly at 130 rpm every 15 s for 30 min at 37°C to complete the gastric digestion phase, and then gastric aliquots were taken. The ileal phase was initiated by the addition of 2 mL 1 M NaHCO_3_ and 5 mL 0.2 M Na maleate buffer (pH 6) to each sample, and the volume was increased to 55 mL with dH_2_O. In quick succession, 0.1 mL of amyloglucosidase and 1 mL of 2% pancreatin solution (in maleate buffer, pH 6) were added to each sample. Samples were then incubated for 120 minutes with constant slow mixing, and aliquots were taken at 20, 60, and 120 minutes during ileal digestion. The tubes were centrifuged (1000 ×g, 2 min) in a Biofuge Primo Centrifuge (Heraeus Instruments, Kendro Laboratory Products, Germany) and an aliquot of the supernatant was removed for analysis of reducing sugars.

#### 2.5.3. Analysis of Reducing Sugars Released during Digestion

Sugar released from the bread during digestion was measured by a colourimetric method adapted from Englyst and Hudson [15] designed to measure monosaccharides after an amyloglucosidase secondary digestion to complete depolymerisation of starch fragments. A total of 0.05 mL of 10 mg/mL glucose standard or sample from the *in vitro* digestion was added to 0.25 mL of enzyme solution A (1% amyloglucosidase in acetate buffer, pH 5.2). Each sample was incubated for 10 minutes at 25°C and then 0.75 mL of 3,5-Dinitrosalicylic acid (DNS) mixture (0.5 mg/mL glucose : 4 M NaOH : DNS reagent mixed in ratio 1 : 1 : 5) was added. The resultant sample was then heated for 15 minutes at 95°C in a water bath. Following this, 3 mL of water was added to each sample which was then left to cool for 20 min in a cold water bath. Absorbance was read at 530 nm on a Shimadzu UV-1201 spectrophotometer (Shimadzu Corporation, Australia) and sugar release was measured in mg per g of bread sample. Slowly digestible starch (SDS) was extrapolated by subtracting the rapidly digestible starch (RDS) measurement at 20 min from the reducing sugars measurement at 120 min during ileal digestion [14].

### 2.6. Statistical Analysis

All experiments were carried out in triplicate and each had a minimum of three replicates for each tea. The data are presented as means (±SEM) and comparisons between samples were carried out by an ANOVA and Tukey's multiple comparison test (SPSS, version 17; SPSS Inc., Chicago, IL, USA). A probability of 5% or less was considered statistically significant.

## 3. Results

### 3.1. Polyphenol Content

Table 1 illustrates that all teas were a significant source of polyphenols. Of the whole teas, “Kagoshima Sencha” brand had a significantly higher polyphenol content (*P* < 0.05) compared to the other commercial whole teas analysed. Of the bagged varieties, green tea infusion had a significantly higher polyphenol content than both white and black teas, as measured by FCR (*P* < 0.05).

### 3.2. Polyphenol Bioaccessibility

The *in vitro* digestion model enables the measurement of polyphenols potentially available for absorption after the gastric and duodenal phases of digestion. Bioaccessibility refers to the proportion of polyphenols which are presented to the brush border for absorption after digestion and gives some indication as to their potential bioavailability *in vivo*.

Table 2 illustrates that the polyphenol content of all tea infusions was enhanced following the gastric digestion phase. This enhancement continued into the duodenal phase, although some tea polyphenols became less bioaccessible relative to the gastric phase.

### 3.3. Sugar Release

**Figure 1:**
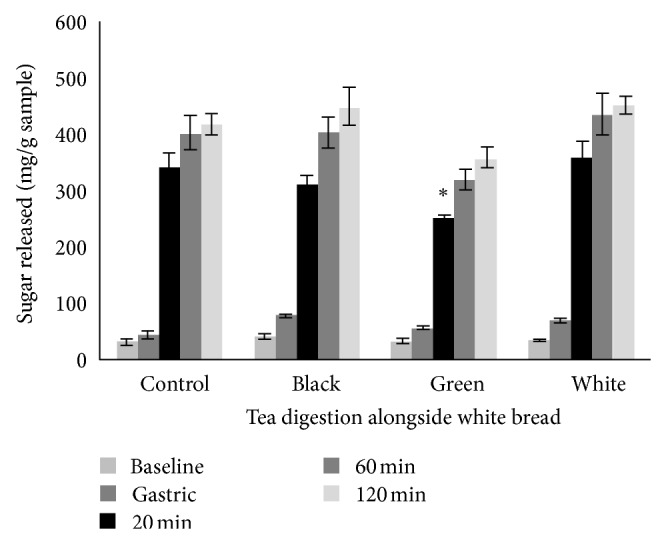
Sugar release from bread samples, when digested alongside either no tea (control), black, green, or white tea. ∗ = significant reduction in sugar release compared to control white bread, *P* < 0.05. Values reported as mg sugar released/g bread sample. Values represent mean  ±  standard error of the means (SEM). 20, 60, and 120 min = stages of intestinal digestion.

At 20 minutes into the duodenal phase of digestion, green tea significantly suppressed RDS release in white bread to 253.83 mg/g bread sample when compared to white tea and the control bread (*P* < 0.05; Figure 1). Black tea showed no significant effect on sugar release at this time point. In all teas, there was a nonsignificant trend to increase SDS release compared to the control.

## 4. Discussion

### 4.1. Polyphenol Content

Of the bagged teas, the black tea had the lowest polyphenol content. The different methods of processing and production impact the polyphenol content of the resultant teas. Total black tea polyphenols decrease during fermentation, and the longer tea is subjected to processing, the lower the polyphenol content [16]. Turkmen and others [17] found that black tea polyphenol content as measured by FCR reached a maximum of 131.9 mg GAE/g tea extract compared to the 87.9 GAE/g in the current study indicating that the polyphenol content of the same tea can vary widely.

The green tea infusion was shown to have more overall reducing power than both black tea and white tea. The production process used in black tea results in the formation of theaflavins. Theaflavins in black tea are dimers of catechins and contain more hydroxyl groups in their structure. In green tea, catechins remain dominant. This could in part explain the differences in reducing potential between black tea and green tea [16].

There has been very little research to date on the polyphenol content and health effects of white tea. Rusak and others [3] found that green tea was a richer source of polyphenols than white tea and the current study supports this. They also found that the concentration of catechins was significantly higher in green tea leaves than white tea leaves.

Ryan and Carolan [18] found that green teas varied in their polyphenol content, ranging from 250 to 750 mg GAE/tea bag. They also found that both the structure of the tea bag and the infusion time influenced the polyphenol content of the teas. FCR values in the current study were slightly lower than those found by Ryan and Carolan [18], averaging 115.5 mg GAE/g tea bag. Rusak and others [3] found that extraction of catechins from green tea was affected by the form of tea used, with extraction from loose green tea leaves being more effective than from refined bagged tea leaves. However, the form of the tea did not affect white tea catechins. Greater tea bag size results in tea solids diffusing into solution faster because of the larger surface area available in which the contents can diffuse [16]. The material of the tea bag can also have an impact on polyphenol diffusion. In the current study, tea bag infusion time and stirring/squeezing of the tea bags were kept constant for all three teas. Tea bags were different weights, the black tea bag weighing more than both white tea and green tea. Weights were, however, corrected for upon calculations.

Of the whole teas analysed, “Kagoshima Sencha” had a significantly higher polyphenol content compared to the other teas. This was the only whole tea that had a higher polyphenol content than any of the bagged teas. Whole teas tend to be more compacted and have undergone much less processing than the bagged tea. Unpublished data from our laboratory indicate that the grinding of leaves to form the bagged teas has the effect of releasing polyphenols and this may in part explain the higher polyphenol content in the bagged teas.

Overall, all teas were shown to be good sources of polyphenols.

### 4.2. Polyphenol Bioaccessibility

In the current study, polyphenol bioaccessibility increased in all teas from baseline to gastric phases suggesting that the polyphenols in these teas may become more available in humans after consumption.

The bioaccessibility increase from baseline to gastric phase was similar for both bagged and whole teas. The compact and relatively unprocessed nature of the whole teas resulted in a lower polyphenol content at baseline. However, the bioaccessibility increases suggest that the digestive enzymes can further release polyphenols from the tea infusion. Green and others [19] looked at the effect of *in vitro* digestion specifically on catechins in green tea and found that catechins had less than 20% recovery after *in vitro *digestion. However, the current study is the first to report and compare the bioaccessibility of total polyphenols from green, white, and black tea infusions. In the current study, with the exception of white bagged tea, the bioaccessibility decreased slightly from gastric to duodenal phases in bagged teas. However, at the duodenal stage values still remained above baseline for all tea varieties. This decrease may be due to the increase in pH during the duodenal phase. Rusak and others [3] showed that the form of tea, either loose or bagged did not affect white tea catechin stability. Therefore, compounds in white tea may be more stable, and thus less susceptible to degradation compared to those in the other teas.

### 4.3. Starch Digestion and Sugar Release

Green tea was the only tea shown to significantly reduce sugar release from white bread. This is a promising finding in that green tea may reduce the RDS of starch rich foods such as bread. However, tea polyphenols have been shown to reduce starch retrogradation. Wu and others [20] found that increased levels of added purified polyphenols (50% EGCG) resulted in decreased retrogradation for resistant starch in rice. From these results, it was predicted that the hydroxyl radical of tea polyphenols combined with rice starch to form hydrogen bonds, preventing the reassociation of the starch chains.

In the current study, white tea had no significant effect on sugar release, although there was a slight increase compared to the control bread. Certain polyphenols or other compounds in white tea may be responsible for interfering with the natural chemical bonds in the bread, therefore rendering the starch more susceptible to degradation. White tea polyphenols were shown to be the most stable throughout digestion, and therefore they may have a greater impact on starch than those polyphenols which are degraded more readily.

Different teas contain different polyphenols, and therefore it is plausible that each tea may have a different effect on sugar release. Teas were used at low concentrations in this study, that is, one tea bag per infusion. Therefore, green tea at low concentrations reduces sugar release from starch samples, whereas white tea at a low dose seems to have the opposite effect. Teas were made as infusions and then added to bread samples at the baseline phase of digestion, whereas other studies have baked tea into breads or looked at the effect of purified tea polyphenol rich extracts. Therefore, different study designs may account for variability in study results. However, what can be seen is that different types of tea do have an effect on starch breakdown and sugar release from breads.

Unpublished data from our laboratory found that glycaemic response (GR) increased over 180 minutes following the consumption of white bread with added black tea extract in comparison to eating white bread alone. A more pronounced effect was also seen when a tea infusion was consumed alongside the white bread and the time taken for this combination to exert its peak GR was prolonged compared to either the control bread or the bread with tea extract added. However, Koh and others [21] found black tea to reduce starch digestion, yet found no effect with green or oolong tea. In the current study, each tea was presented as an infusion alongside bread during digestion and the black tea infusion had no significant effect on sugar release from bread. The form in which the black tea is used, the preparation method, and the concentration used may all be factors to account for the variation in results between studies.

Deshpande and Salunkhe [22] found that in isolation tannic acid and catechins decreased *in vitro *digestibility of various types of starch sources. Also, Bjork and Nyman [23] found that phytic acid and tannic acid reduced starch hydrolysis in the digestive tract. However, isolated polyphenols may have a different effect on starch digestion compared to phenolics in combination. For example, green tea is one of the richest sources of phenolic compounds and includes catechins such as EGCG, procyanidins, and quercetin [24]. Therefore, a combination of the phenolics in tea may have synergistic effects, either enhancing or reducing the degree of starch breakdown and sugar release compared to isolated catechins or tannins [25, 26]. The reason for the reduction in sugar release seen in the current study may be because of the structural bonding of the green tea polyphenols with starch molecules. Both black tea and green tea contain conjugate forms of catechins, and these compounds may interfere with the way in which starch is broken down. Therefore, future research could look into accessing the polyphenol profile of the different teas to determine which polyphenols have an effect on reducing starch breakdown.

Tea polyphenols may have an inhibitory effect on digestive enzymes such as *α*-amylase and *α*-glucosidase. Black tea and to a lesser extent green tea were shown to inhibit *α*-amylase in human saliva and removal of tea tannins resulted in loss of the inhibitory activity [27]. Hara and Honda [28] found that both catechins and theaflavins inhibited salivary *α*-amylase and whilst, various teas differed in the extent of inhibitory activity, black tea showed consistently greater inhibitory activity. Kwon and others [29] found that black tea and white tea showed almost a 40% inhibition of *α*-amylase, with green tea showing only a 30% inhibition. They also found black and white tea to have a higher *α*-glucosidase inhibitory activity than green and oolong teas. At higher concentrations of polyphenols, {50 *μ*g GAE/mL, 100 *μ*g GAE/mL and 200 *μ*g GAE/mL}  *α*-glucosidase inhibition was increased in all teas. Although enzyme inhibition was not tested in the current study, a future area of study is to investigate the effect of tea polyphenols on digestive enzymes.

Finally, it should be noted that many studies evaluating the GR to foods contain the addition of tea or coffee in their test meals. Based on the current results and on previous studies, the polyphenols in tea may affect BG, thus affecting the results of the test foods studied. It is important that when testing foods for different effects on disease parameters, the combination of ingredients is considered. A single food in isolation may have different effects on GR than that food with an additional tea beverage.

## Figures and Tables

**Table 1:** Polyphenol content (expressed as gallic acid equivalents (GAEs) per gram and per serving (3 g in 200 mL water)).

Tea	GAE (mg/g Tea)	GAE (mg/serving)
Jing Assam Breakfast^1^	48.6	145.9
Organic Jade Sword^1^	42.6	127.9
Organic Dragon Well^1^	54.9	164.6
Jing Earl Grey^1^	62.2	186.7
Jasmine Pearls^1^	23.3	69.9
Flowering Osmanthus^1^	10.3	75.2
Flowering Jasmine and Lily^1^	13.8	96.3
Tieguanyin^1^	28.5	85.5
Moroccan Mint^1^	48.7	146.2
Jing Ceylon^1^	58.7	176.0
Jing Darjeeling 2nd Flush^1^	47.3	141.8
Jasmine Silver Needle^1^	20.4	61.1
Yellow Gold Oolong^1^	23.5	70.5
Jun Shan Silver Needle^1^	38.6	115.7
Kagoshima Sencha^1^	95.3^a^	285.8
Taiwan Ali Shan Oolong^1^	20.1	60.3
Clipper Black Tea^2^	87.9	263.7
Clipper Green Tea^2^	115.5^b^	346.5
Clipper White Tea^2^	102.8	308.4

^1^Whole teas, ^2^bagged teas.

^a^significantly (*P* < 0.05) greater than all other whole tea samples.

^b^significantly (*P* < 0.05) greater than all other bagged tea samples.

**Table 2:** % Bioaccessibility of the polyphenol content after the gastric and duodenal phases of digestion.

Tea	Gastric (%)	Duodenal (%)
Jing Assam Breakfast^1^	140.7	121.2
Organic Jade Sword^1^	133.2	131.0
Organic Dragon Well^1^	123.2	128.1
Jing Earl Grey^1^	121.0	127.7
Jasmine Pearls^1^	172.2	204.0
Flowering Osmanthus^1^	176.8	189.3
Flowering Jasmine and Lily^1^	160.5	174.6
Tieguanyin^1^	161.7	185.2
Moroccan Mint^1^	128.0	124.3
Jing Ceylon^1^	124.6	127.4
Jing Darjeeling 2nd flush^1^	133.5	142.4
Jasmine Silver Needle^1^	204.1	233.6
Yellow Gold Oolong^1^	194.4	233.5
Jun Shan Silver Needle^1^	153.9	147.1
Kagoshima Sencha^1^	143.7	134.7
Taiwan Ali Shan Oolong^1^	207.9	231.1
Clipper Black Tea^2^	136.6	126.6
Clipper Green Tea^2^	132.0	125.3
Clipper White Tea^2^	165.1	176.6

^1^Whole teas, ^2^bagged teas.
